# 3,4,5-Trihydr­oxy-*N*′-(2-hydr­oxy-5-nitro­benzyl­idene)benzohydrazide mono­hydrate

**DOI:** 10.1107/S1600536809010563

**Published:** 2009-03-28

**Authors:** Abeer A. Abdul Alhadi, Hapipah Mohd. Ali, Seik Weng Ng

**Affiliations:** a Department of Chemistry, University of Malaya, 50603 Kuala Lumpur, Malaysia

## Abstract

The benzohydrazide mol­ecule of the title compound, C_14_H_11_N_3_O_7_·H_2_O, is planar (r.m.s. deviation = 0.068 Å). The benzohydrazide mol­ecule and the uncoordinated water mol­ecule inter­act through O—H⋯O hydrogen bonds; these together with O—H⋯N and N—H⋯O hydrogen bonds form a three-dimensional network.

## Related literature

For the the parent *N*′-(2-hydroxy­benzyl­idene)benzohydrazide, see: Lyubchova *et al.* (1995 ▶). For other *N*′-(2-hydr­oxy-5-nitro­benzyl­idene)benzohydrazides, see: Ali *et al.* (2005 ▶); Lyubchova *et al.* (1995 ▶); Xu & Liu (2006 ▶).
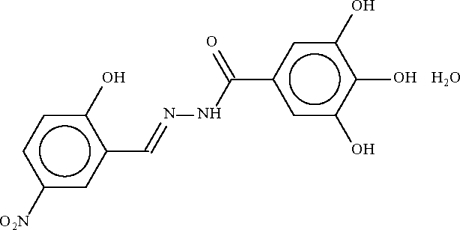


## Experimental

### 

#### Crystal data

C_14_H_11_N_3_O_7_·H_2_O
*M* *_r_* = 351.27
Triclinic, 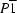

*a* = 7.0097 (2) Å
*b* = 7.8380 (2) Å
*c* = 13.2953 (3) Å
α = 75.597 (1)°
β = 88.826 (2)°
γ = 81.929 (2)°
*V* = 700.42 (3) Å^3^
*Z* = 2
Mo *K*α radiation
μ = 0.14 mm^−1^
*T* = 123 K
0.15 × 0.10 × 0.02 mm

#### Data collection

Bruker SMART APEX diffractometer
Absorption correction: none
6629 measured reflections
3209 independent reflections
2373 reflections with *I* > 2σ(*I*)
*R*_int_ = 0.019

#### Refinement

*R*[*F* ^2^ > 2σ(*F* ^2^)] = 0.038
*wR*(*F* ^2^) = 0.112
*S* = 1.03
3209 reflections
254 parameters
7 restraints
H atoms treated by a mixture of independent and constrained refinement
Δρ_max_ = 0.42 e Å^−3^
Δρ_min_ = −0.25 e Å^−3^

### 

Data collection: *APEX2* (Bruker, 2008 ▶); cell refinement: *SAINT* (Bruker, 2008 ▶); data reduction: *SAINT*; program(s) used to solve structure: *SHELXS97* (Sheldrick, 2008 ▶); program(s) used to refine structure: *SHELXL97* (Sheldrick, 2008 ▶); molecular graphics: *X-SEED* (Barbour, 2001 ▶); software used to prepare material for publication: *publCIF* (Westrip, 2009 ▶).

## Supplementary Material

Crystal structure: contains datablocks global, I. DOI: 10.1107/S1600536809010563/tk2399sup1.cif

Structure factors: contains datablocks I. DOI: 10.1107/S1600536809010563/tk2399Isup2.hkl

Additional supplementary materials:  crystallographic information; 3D view; checkCIF report

## Figures and Tables

**Table 1:** Hydrogen-bond geometry (Å, °)

*D*—H⋯*A*	*D*—H	H⋯*A*	*D*⋯*A*	*D*—H⋯*A*
O1—H1⋯N2	0.84 (1)	1.81 (1)	2.581 (2)	152 (2)
O5—H5⋯O6^i^	0.84 (1)	2.16 (2)	2.847 (2)	139 (2)
O6—H6⋯O1w^ii^	0.84 (1)	1.81 (1)	2.630 (2)	162 (2)
O7—H7⋯O4^iii^	0.84 (1)	1.87 (1)	2.715 (2)	177 (2)
O1w—H11⋯O5	0.84 (1)	2.13 (1)	2.918 (2)	156 (2)
O1w—H12⋯O1^iv^	0.84 (1)	2.13 (1)	2.962 (2)	169 (2)
N3—H3⋯O3^v^	0.88 (1)	2.07 (1)	2.890 (2)	155 (2)

Symmetry codes: (i) 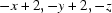; (ii) 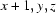; (iii) 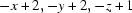; (iv) 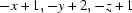; (v) 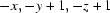.

**Fig. 1.**
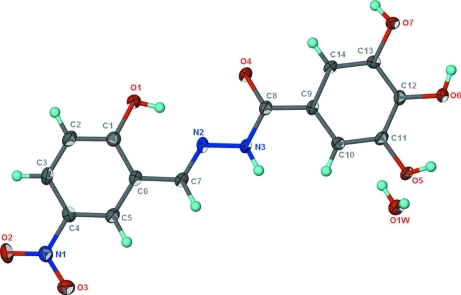
Thermal ellipsoid plot (Barbour, 2001) of C14H11N3O7.H2O at the 70% probability level; hydrogen atoms are drawn as spheres of arbitrary radius.

## supplementary crystallographic information

### Experimental

5-Nitro-2-hydroxybenzaldehyde (0.33 g, 2 mmol) and 3,4,5-trihydroxybenzoylhydrazide (0.36 g, 2 mmol) were heated in ethanol (50 ml) for several hours. The solvent was removed and the product recrystallized from DMSO.

### Refinement

Carbon-bound H-atoms were placed in calculated positions [C—H 0.95 Å, *U*(H) = 1.2*U*(C)], and were included in the refinement in the riding model approximation.

The amino and hydroxy H-atoms were located in a difference Fourier map, and were refined with distance restraints of N–H 0.88±0.01 Å and O–H 0.84±0.01 Å, respectively; their temperature factors were refined isotropically.

### Crystal data

**Table:**

C_14_H_11_N_3_O_7_·H_2_O	*Z* = 2
*M**_r_* = 351.27	*F*(000) = 364
Triclinic, *P*1	*D*_x_ = 1.666 Mg m^−^^3^
Hall symbol: -P 1	Mo *K*α radiation, λ = 0.71073 Å
*a* = 7.0097 (2) Å	Cell parameters from 2084 reflections
*b* = 7.8380 (2) Å	θ = 2.7–28.3°
*c* = 13.2953 (3) Å	µ = 0.14 mm^−^^1^
α = 75.597 (1)°	*T* = 123 K
β = 88.826 (2)°	Plate, yellow
γ = 81.929 (2)°	0.15 × 0.10 × 0.02 mm
*V* = 700.42 (3) Å^3^	

### Data collection

**Table:**

Bruker SMART APEX diffractometer	2373 reflections with *I* > 2σ(*I*)
Radiation source: fine-focus sealed tube	*R*_int_ = 0.019
graphite	θ_max_ = 27.5°, θ_min_ = 1.6°
ω scans	*h* = −9→9
6629 measured reflections	*k* = −10→10
3209 independent reflections	*l* = −17→16

### Refinement

**Table:**

Refinement on *F*^2^	Primary atom site location: structure-invariant direct methods
Least-squares matrix: full	Secondary atom site location: difference Fourier map
*R*[*F*^2^ > 2σ(*F*^2^)] = 0.038	Hydrogen site location: inferred from neighbouring sites
*wR*(*F*^2^) = 0.112	H atoms treated by a mixture of independent and constrained refinement
*S* = 1.03	*w* = 1/[σ^2^(*F*_o_^2^) + (0.0583*P*)^2^ + 0.1907*P*] where *P* = (*F*_o_^2^ + 2*F*_c_^2^)/3
3209 reflections	(Δ/σ)_max_ = 0.001
254 parameters	Δρ_max_ = 0.42 e Å^−^^3^
7 restraints	Δρ_min_ = −0.25 e Å^−^^3^

### Fractional atomic coordinates and isotropic or equivalent isotropic displacement parameters (Å^2^)

**Table:**

	*x*	*y*	*z*	*U*_iso_*/*U*_eq_	
O1	0.45792 (16)	0.65420 (15)	0.74975 (9)	0.0199 (3)	
O2	−0.28084 (18)	0.28619 (17)	0.85473 (9)	0.0280 (3)	
O3	−0.29787 (16)	0.35460 (16)	0.68665 (9)	0.0235 (3)	
O4	0.75724 (17)	0.84619 (16)	0.53567 (9)	0.0241 (3)	
O5	0.70666 (16)	1.00100 (17)	0.07165 (8)	0.0216 (3)	
O6	1.05293 (16)	1.11263 (16)	0.08126 (8)	0.0195 (3)	
O7	1.21628 (16)	1.10003 (17)	0.27137 (9)	0.0216 (3)	
O1W	0.36719 (17)	1.26409 (18)	0.06982 (9)	0.0236 (3)	
N1	−0.21856 (19)	0.35371 (18)	0.76939 (10)	0.0188 (3)	
N2	0.43352 (19)	0.71596 (18)	0.55014 (10)	0.0174 (3)	
N3	0.50987 (19)	0.77875 (19)	0.45456 (10)	0.0182 (3)	
C1	0.2933 (2)	0.5835 (2)	0.75146 (12)	0.0170 (3)	
C2	0.2175 (2)	0.5116 (2)	0.84825 (12)	0.0206 (3)	
H2	0.2824	0.5136	0.9099	0.025*	
C3	0.0492 (2)	0.4379 (2)	0.85482 (12)	0.0196 (3)	
H3A	−0.0027	0.3890	0.9206	0.024*	
C4	−0.0440 (2)	0.4361 (2)	0.76366 (12)	0.0173 (3)	
C5	0.0277 (2)	0.5064 (2)	0.66704 (12)	0.0172 (3)	
H5A	−0.0392	0.5042	0.6060	0.021*	
C6	0.1980 (2)	0.5805 (2)	0.65926 (12)	0.0166 (3)	
C7	0.2744 (2)	0.6509 (2)	0.55666 (12)	0.0179 (3)	
H7A	0.2075	0.6485	0.4957	0.021*	
C8	0.6794 (2)	0.8441 (2)	0.45388 (12)	0.0158 (3)	
C9	0.7687 (2)	0.9115 (2)	0.35203 (11)	0.0152 (3)	
C10	0.6888 (2)	0.9198 (2)	0.25525 (12)	0.0157 (3)	
H10	0.5683	0.8798	0.2504	0.019*	
C11	0.7882 (2)	0.9876 (2)	0.16644 (11)	0.0160 (3)	
C12	0.9662 (2)	1.0473 (2)	0.17210 (12)	0.0154 (3)	
C13	1.0437 (2)	1.0389 (2)	0.26925 (12)	0.0159 (3)	
C14	0.9458 (2)	0.9706 (2)	0.35795 (12)	0.0164 (3)	
H14	0.9998	0.9637	0.4240	0.020*	
H1	0.482 (3)	0.691 (3)	0.6866 (9)	0.043 (7)*	
H5	0.789 (3)	1.020 (3)	0.0256 (16)	0.060 (8)*	
H6	1.151 (2)	1.158 (3)	0.0912 (18)	0.045 (7)*	
H7	1.228 (3)	1.117 (3)	0.3308 (10)	0.045 (7)*	
H11	0.452 (3)	1.191 (3)	0.0523 (19)	0.047 (7)*	
H12	0.408 (3)	1.278 (3)	0.1260 (12)	0.055 (8)*	
H3	0.453 (3)	0.769 (2)	0.3981 (10)	0.022 (5)*	

### Atomic displacement parameters (Å^2^)

**Table:**

	*U*^11^	*U*^22^	*U*^33^	*U*^12^	*U*^13^	*U*^23^
O1	0.0212 (6)	0.0250 (6)	0.0149 (6)	−0.0097 (5)	−0.0003 (5)	−0.0041 (5)
O2	0.0256 (6)	0.0346 (7)	0.0205 (6)	−0.0096 (5)	0.0064 (5)	0.0017 (5)
O3	0.0212 (6)	0.0307 (7)	0.0219 (6)	−0.0077 (5)	0.0003 (5)	−0.0102 (5)
O4	0.0268 (6)	0.0361 (7)	0.0116 (5)	−0.0148 (5)	0.0000 (5)	−0.0040 (5)
O5	0.0172 (6)	0.0399 (7)	0.0094 (5)	−0.0089 (5)	0.0009 (4)	−0.0067 (5)
O6	0.0184 (6)	0.0301 (7)	0.0117 (5)	−0.0098 (5)	0.0032 (4)	−0.0053 (5)
O7	0.0186 (6)	0.0368 (7)	0.0134 (6)	−0.0132 (5)	0.0016 (5)	−0.0087 (5)
O1W	0.0215 (6)	0.0334 (7)	0.0169 (6)	−0.0077 (5)	0.0004 (5)	−0.0060 (5)
N1	0.0174 (7)	0.0206 (7)	0.0181 (7)	−0.0030 (5)	0.0028 (5)	−0.0041 (6)
N2	0.0194 (7)	0.0200 (7)	0.0123 (6)	−0.0046 (5)	0.0033 (5)	−0.0023 (5)
N3	0.0186 (7)	0.0271 (8)	0.0095 (6)	−0.0077 (6)	0.0015 (5)	−0.0034 (5)
C1	0.0184 (7)	0.0166 (8)	0.0163 (8)	−0.0028 (6)	0.0003 (6)	−0.0045 (6)
C2	0.0251 (8)	0.0233 (9)	0.0133 (8)	−0.0045 (7)	−0.0013 (6)	−0.0038 (6)
C3	0.0241 (8)	0.0213 (8)	0.0124 (7)	−0.0042 (7)	0.0032 (6)	−0.0016 (6)
C4	0.0176 (7)	0.0166 (8)	0.0173 (8)	−0.0031 (6)	0.0027 (6)	−0.0036 (6)
C5	0.0185 (7)	0.0192 (8)	0.0135 (7)	−0.0019 (6)	−0.0007 (6)	−0.0036 (6)
C6	0.0181 (8)	0.0178 (8)	0.0134 (7)	−0.0024 (6)	0.0009 (6)	−0.0032 (6)
C7	0.0202 (8)	0.0215 (8)	0.0124 (7)	−0.0043 (6)	−0.0001 (6)	−0.0044 (6)
C8	0.0177 (7)	0.0169 (8)	0.0127 (7)	−0.0029 (6)	0.0005 (6)	−0.0034 (6)
C9	0.0174 (7)	0.0170 (8)	0.0110 (7)	−0.0024 (6)	0.0022 (6)	−0.0034 (6)
C10	0.0131 (7)	0.0202 (8)	0.0145 (7)	−0.0031 (6)	0.0008 (6)	−0.0051 (6)
C11	0.0166 (7)	0.0207 (8)	0.0112 (7)	−0.0014 (6)	−0.0002 (6)	−0.0056 (6)
C12	0.0154 (7)	0.0183 (8)	0.0121 (7)	−0.0024 (6)	0.0028 (6)	−0.0034 (6)
C13	0.0143 (7)	0.0196 (8)	0.0147 (7)	−0.0045 (6)	0.0004 (6)	−0.0048 (6)
C14	0.0185 (7)	0.0198 (8)	0.0116 (7)	−0.0039 (6)	−0.0018 (6)	−0.0043 (6)

### Geometric parameters (Å, °)

**Table:**

O1—C1	1.3456 (19)	C1—C6	1.415 (2)
O1—H1	0.840 (10)	C2—C3	1.377 (2)
O2—N1	1.2263 (17)	C2—H2	0.9500
O3—N1	1.2408 (17)	C3—C4	1.393 (2)
O4—C8	1.2313 (18)	C3—H3A	0.9500
O5—C11	1.3684 (18)	C4—C5	1.380 (2)
O5—H5	0.835 (10)	C5—C6	1.391 (2)
O6—C12	1.3567 (18)	C5—H5A	0.9500
O6—H6	0.844 (10)	C6—C7	1.460 (2)
O7—C13	1.3654 (18)	C7—H7A	0.9500
O7—H7	0.842 (10)	C8—C9	1.486 (2)
O1W—H11	0.837 (10)	C9—C14	1.394 (2)
O1W—H12	0.843 (10)	C9—C10	1.397 (2)
N1—C4	1.454 (2)	C10—C11	1.386 (2)
N2—C7	1.283 (2)	C10—H10	0.9500
N2—N3	1.3705 (18)	C11—C12	1.402 (2)
N3—C8	1.358 (2)	C12—C13	1.394 (2)
N3—H3	0.882 (9)	C13—C14	1.380 (2)
C1—C2	1.397 (2)	C14—H14	0.9500
			
C1—O1—H1	105.4 (16)	C5—C6—C1	118.80 (14)
C11—O5—H5	109.5 (19)	C5—C6—C7	119.23 (14)
C12—O6—H6	111.2 (16)	C1—C6—C7	121.96 (14)
C13—O7—H7	106.8 (16)	N2—C7—C6	118.88 (14)
H11—O1W—H12	105 (2)	N2—C7—H7A	120.6
O2—N1—O3	122.81 (13)	C6—C7—H7A	120.6
O2—N1—C4	119.27 (13)	O4—C8—N3	120.72 (14)
O3—N1—C4	117.92 (13)	O4—C8—C9	121.07 (14)
C7—N2—N3	119.72 (13)	N3—C8—C9	118.21 (13)
C8—N3—N2	116.36 (13)	C14—C9—C10	119.94 (13)
C8—N3—H3	123.5 (13)	C14—C9—C8	114.80 (13)
N2—N3—H3	120.0 (13)	C10—C9—C8	125.25 (14)
O1—C1—C2	117.78 (14)	C11—C10—C9	118.85 (14)
O1—C1—C6	122.00 (14)	C11—C10—H10	120.6
C2—C1—C6	120.21 (14)	C9—C10—H10	120.6
C3—C2—C1	120.36 (15)	O5—C11—C10	118.74 (13)
C3—C2—H2	119.8	O5—C11—C12	119.86 (13)
C1—C2—H2	119.8	C10—C11—C12	121.36 (14)
C2—C3—C4	119.02 (14)	O6—C12—C13	123.45 (13)
C2—C3—H3A	120.5	O6—C12—C11	117.42 (13)
C4—C3—H3A	120.5	C13—C12—C11	119.13 (13)
C5—C4—C3	121.78 (14)	O7—C13—C14	122.96 (14)
C5—C4—N1	118.55 (14)	O7—C13—C12	117.31 (13)
C3—C4—N1	119.64 (14)	C14—C13—C12	119.73 (14)
C4—C5—C6	119.81 (14)	C13—C14—C9	120.99 (14)
C4—C5—H5A	120.1	C13—C14—H14	119.5
C6—C5—H5A	120.1	C9—C14—H14	119.5
			
C7—N2—N3—C8	−179.41 (15)	N2—N3—C8—C9	179.23 (13)
O1—C1—C2—C3	179.85 (15)	O4—C8—C9—C14	1.7 (2)
C6—C1—C2—C3	−0.3 (2)	N3—C8—C9—C14	−177.85 (14)
C1—C2—C3—C4	0.0 (3)	O4—C8—C9—C10	−177.86 (16)
C2—C3—C4—C5	−0.1 (3)	N3—C8—C9—C10	2.6 (2)
C2—C3—C4—N1	178.09 (15)	C14—C9—C10—C11	−0.1 (2)
O2—N1—C4—C5	177.95 (14)	C8—C9—C10—C11	179.45 (15)
O3—N1—C4—C5	−1.8 (2)	C9—C10—C11—O5	−177.89 (14)
O2—N1—C4—C3	−0.3 (2)	C9—C10—C11—C12	0.0 (2)
O3—N1—C4—C3	179.95 (14)	O5—C11—C12—O6	−1.7 (2)
C3—C4—C5—C6	0.4 (2)	C10—C11—C12—O6	−179.59 (14)
N1—C4—C5—C6	−177.79 (14)	O5—C11—C12—C13	177.58 (14)
C4—C5—C6—C1	−0.7 (2)	C10—C11—C12—C13	−0.3 (2)
C4—C5—C6—C7	178.65 (14)	O6—C12—C13—O7	−0.1 (2)
O1—C1—C6—C5	−179.54 (14)	C11—C12—C13—O7	−179.35 (14)
C2—C1—C6—C5	0.6 (2)	O6—C12—C13—C14	179.91 (15)
O1—C1—C6—C7	1.2 (2)	C11—C12—C13—C14	0.7 (2)
C2—C1—C6—C7	−178.71 (15)	O7—C13—C14—C9	179.26 (15)
N3—N2—C7—C6	178.44 (14)	C12—C13—C14—C9	−0.8 (2)
C5—C6—C7—N2	−178.70 (15)	C10—C9—C14—C13	0.5 (2)
C1—C6—C7—N2	0.6 (2)	C8—C9—C14—C13	−179.11 (14)
N2—N3—C8—O4	−0.3 (2)		

### Hydrogen-bond geometry (Å, °)

**Table:**

*D*—H···*A*	*D*—H	H···*A*	*D*···*A*	*D*—H···*A*
O1—H1···N2	0.84 (1)	1.81 (1)	2.581 (2)	152 (2)
O5—H5···O6^i^	0.84 (1)	2.16 (2)	2.847 (2)	139 (2)
O6—H6···O1w^ii^	0.84 (1)	1.81 (1)	2.630 (2)	162 (2)
O7—H7···O4^iii^	0.84 (1)	1.87 (1)	2.715 (2)	177 (2)
O1w—H11···O5	0.84 (1)	2.13 (1)	2.918 (2)	156 (2)
O1w—H12···O1^iv^	0.84 (1)	2.13 (1)	2.962 (2)	169 (2)
N3—H3···O3^v^	0.88 (1)	2.07 (1)	2.890 (2)	155 (2)

Symmetry codes: (i) −*x*+2, −*y*+2, −*z*; (ii) *x*+1, *y*, *z*; (iii) −*x*+2, −*y*+2, −*z*+1; (iv) −*x*+1, −*y*+2, −*z*+1; (v) −*x*, −*y*+1, −*z*+1.
